# Assessment of Posterior Dentoalveolar Expansion with Invisalign in Adult Patients

**DOI:** 10.3390/ijerph20054318

**Published:** 2023-02-28

**Authors:** Vincent Santucci, Paul Emile Rossouw, Dimitrios Michelogiannakis, Tarek El-Baily, Changyong Feng

**Affiliations:** 1 Private Practice, 1700 Waterfront Building 700, Wichita, KS 67206, USA; santucci1211@gmail.com; 2 Division of Orthodontics and Dentofacial Orthopedics, Eastman Institute for Oral Health, University of Rochester, 625 Elmwood Ave., Rochester, NY 14620, USA; dimitrios_michelogiannakis@urmc.rochester.edu; 3 7-020D Katz Group Centre for Pharmacy and Health Research, Department of Dentistry and Dental Hygiene, University of Alberta, Edmonton, AB T6G 2R7, Canada; telbaily@ualberta.ca; 4 Medical Center, Department of Biostatistics and Computational Biology, School of Medicine and Dentistry, University of Rochester, Rochester, NY 14642, USA; changyong_feng@urmc.rochester.edu

**Keywords:** orthodontic expansion, clear aligner (Invisalign), prediction, overestimation

## Abstract

Purpose: The primary aim was to evaluate dentoalveolar expansion with Invisalign clear aligners comparing linear measurements in ClinCheck vs. cone beam computed tomography (CBCT). This would enable an assessment of to what extent expansion gained from Invisalign clear aligners was due to buccal tipping and/or bodily translation of the posterior teeth. The study also evaluated the predictive value of Invisalign ClinCheck^®^ (Align Technology, San Jose, CA, USA) to final outcomes. Methods: The orthodontic records of thirty-two (32) subjects comprised the sample to conduct this study. Linear values of the upper arch width were measured for premolars and molars at two different points (occlusal and gingival) utilized for ClinCheck^®^ measurements and three different points for CBCT measurements before (T_0_ and after treatment (T_1_). Paired T-tests at a significance level of 0.05 were used for analyses. Results: Expansion was found to be possible with Invisalign clear aligners. However, more expansion was measured at the cusp tips compared to gingival margins (*p* < 0.0001), indicating more tipping was occurring than bodily translation. ClinCheck^®^ also showed a significant overestimation of the amount of expansion capable, with nearly 70% expression in the first premolar area, and the expression decreased as one moved posteriorly with only 35% expressed at the first molar area (*p* < 0.0001). Conclusions: Dentoalveolar expansion with Invisalign is achieved through buccal tipping of posterior teeth and bodily translation; and there is a significant overestimation of the amount of expansion achieved between ClinCheck^®^ and clinical results.

## 1. Introduction

Align Technology introduced the Invisalign clear aligners appliance in 1999 as a novel method of straightening teeth without braces [1]. Since then, Invisalign has made great progress in terms of treatment planning methods, materials, manufacturing, and marketing. The company’s powerful marketing has increased the public’s demand for clear aligners to the point where Invisalign is an essential part of most orthodontic practices today. Much speculation exists as to the use of clear aligners in orthodontics. Moreover, there is no strong evidence regarding the capabilities and/or limitations of clear aligners [2].

Several methods can be used to assess the quality of Invisalign treatment, including the American Board of Orthodontics cast and radiograph system, the Peer Assessment Rating (PAR) scores, and other orthodontic objective occlusal criteria. The most notable conclusions were that Invisalign is not as effective as fixed appliances for expansion [2], it seems to cause more relapse, [3] and it is not very effective in controlling buccolingual inclinations, [4,5,6] occlusal contacts, occlusal relationships, overjet [4], and overbite [7]. These objective methods of assessing treatment outcomes have some limitations and do not explain the etiology of unsatisfactory results in extensive detail [6].

Dental arch expansion has been reported to be possible with Invisalign clear aligners and may be required as a method to improve esthetics of the smile by broadening the dental arches. This protocol has been suggested for use to create space in order to resolve irregular alignment of teeth (crowding) as well as a way of correcting dentoalveolar posterior crossbites. In treatment of complex malocclusions using Invisalign clear aligners, it has been reported that buccal expansion of the dental arches can be attained to eliminate crowding and modify the arch form to appropriate alignment [8]. The range of expansion was said to be 2–4 mm. Dentoalveolar expansion has been shown to be possible with Invisalign clear aligners and can be an alternative to interproximal enamel reduction (IPR) [9]. Expansion of the dental arches should be limited to 2–3 mm of arch width per quadrant to reduce relapse of the expanded arch and as well as avoiding gingival recession [9]. Expansion can be an indication to use Invisalign clear aligners when there is a need to resolve 1–5 mm of crowding or make space for a blocked-out tooth [10].

Present technologies and incorporation of 3-dimensional scanning have provided new methods to evaluate the accuracy of Invisalign through superimposing predicted and attained 3D models. A few studies have used 3D superimposition to measure the accuracy of different types of tooth movements, but the results have been unclear [11,12]. However, 3D superimposition is limited by the lack of stable anatomic structures on the predicted models due to ClinCheck^®^ (Align Technology) only presenting clinical crowns and virtual gingiva. Similar studies may be able to provide useful information for efficient treatment planning with ClinCheck^®^. For example, if accurate data of a specific tooth movement are known, overcorrecting can be planned, or staging movements can be accomplished in smaller increments to lead to the ideal outcome. Moreover, limited data exist with respect to the amount of discrepancy between predicted and actual attained movements with Invisalign. Mean accuracy of tooth movement in the anterior region was observed to be 41% with Invisalign clear aligners [12]. An internal study from Align Technology reported that about 80% of the tooth movements seen on ClinCheck^®^ can be expected during treatment with clear aligners.

Previous studies have provided useful clinical data. However, much is to be learned about the biomechanics and limitations of clear aligners. According to recent systematic reviews, the quality of available studies did not allow providing evidence-based conclusions [13]. It thus appears that information reported about Invisalign clear aligner use is still based on clinical experience rather than scientific evidence [12].

Given the limited information in the literature [14], especially regarding posterior transverse changes, the primary aim was to evaluate dentoalveolar expansion with Invisalign clear aligners comparing linear measurements in ClinCheck vs. cone beam computed tomography (CBCT). The changes in tooth movement during the expansion would enable one to determine whether dental arch expansion gained from Invisalign clear aligners is due to true dentoalveolar expansion (bodily movement of posterior teeth) or due to buccal tipping of posterior teeth crowns. This study also looked at the predictive value of Invisalign ClinCheck^®^ compared to the final outcome to enable the clinician to anticipate incorporating overcorrection as part of the treatment plan, thereby reducing extensive modifications, such as refinements, mid-course correction, and treatment time.

## 2. Materials and Methods

The study was reviewed and approved for Ethical and Scientific requirements by the IRB, University of Rochester (IRB#00003823). The ClinCheck^®^ (Align Technology, San Jose, CA, USA) and cone beam computed tomography (CBCT) records of thirty-two (32) subjects comprised the sample to conduct this study. Utilizing descriptions from previous studies examining expansion through CBCT analysis, sufficient sample size was calculated by a power analysis to report effect size, power of outcome (80%) at a significance level of *p* < 0.05 to produce clinically meaningful scientific and statistical results [15]. Subjects who received expansion treatment between December 2014 and December 2018 were identified by the solo private practitioner who performed the treatment. Subjects were de-identified according to HIPAA requirements, and the orthodontic records were provided to the investigators to assess the Invisalign clear aligner treatment effects. The average age was 29.25 ± 9.75 years, with a male to female distribution of 13 to 19, respectively. No restrictions were placed on the racial or ethnic distribution of subjects.

Subjects who met the eligibility criteria were enrolled consecutively. Records were obtained from subjects who fell into our inclusion/exclusion criteria:

### 2.1. Inclusion Criteria

- Adult patients ≥ 18 years and exhibiting a dental Class II division 1 malocclusion with crowding
- Non-extraction treatment
- Treated without any expansion auxiliaries (only Invisalign clear aligners treatment with attachments)
- CBCT of adequate quality available before and after treatment
- Those with acceptable compliance to aligner prescription
- Patients who needed transverse expansion for ideal treatment

### 2.2. Exclusion Criteria

- Patients younger than ≤18
- Measurements with a missing contralateral tooth
- Refinement treatments
- Patients with severe systemic diseases/conditions
- Patients treated with auxiliaries outside of Invisalign attachments
- Patients who need interproximal reduction (IPR) distal to canines
- Any patient needing midcourse correction

**Figure 1:**
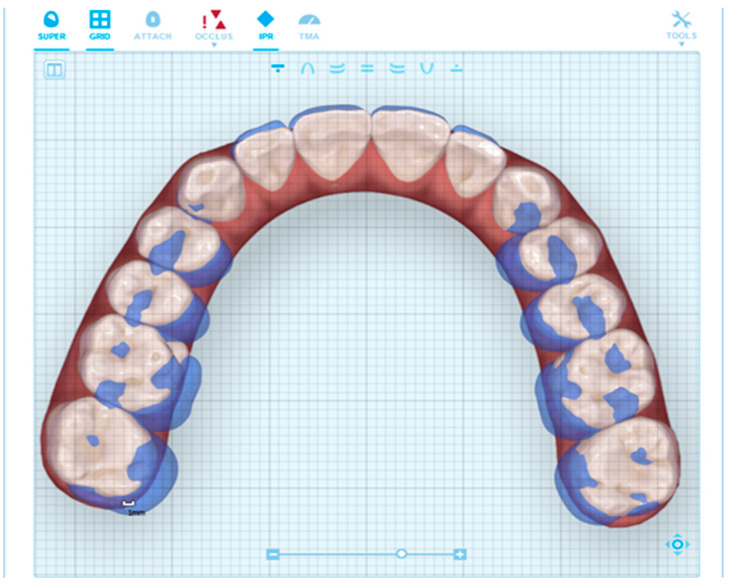
Maxillary dental arch in the ClinCheck^®^ mode showing superimposition of the expansion changes (T_0_–T_1_); blue, representing the original arch form and white, the expansion predicted outcome. Two arch width measurements were obtained between the lingual cusp tip and gingival margin (Align Technology, San Jose, CA, USA).

Linear measurements were compared at the initiation of treatment (T_0_) and the completion of treatment (T_1_) to measure the outcomes of treatment in the transverse dimension (Figure 1). Similar measuring techniques were followed by Charalampakis et al. [16] and Sousa et al. [17]. The time between T_0_ and T_1_ varied based on treatment times to correct malocclusion but ranged from 1.5 to 3 years.

**Figure 2:**
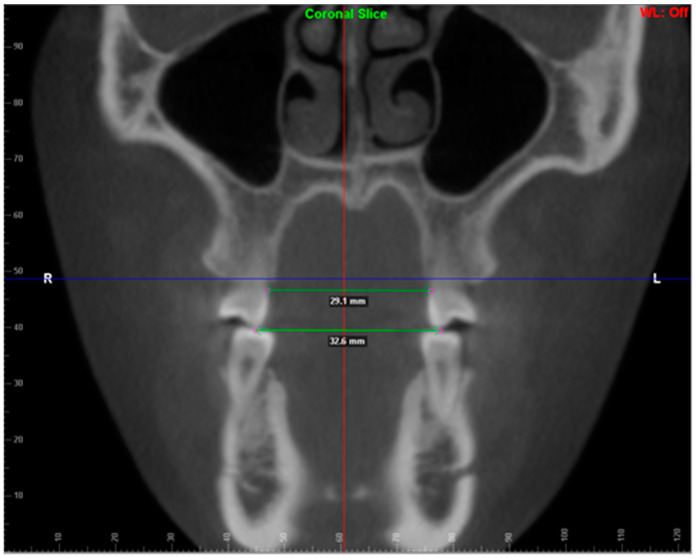
CBCT transverse sectional image shows the transverse measurements at the maxillary first molar level; from the lingual cemento-enamel junction (gingival margin) and between the tip of the lingual cusp.

Linear measurements of the maxillary dental arch were obtained for premolars and permanent first molars. ClinCheck^®^ measurements were made at two different points for each tooth, lingual cusp tip and gingival margin. ClinCheck^®^ measurements were made on the Invisalign ClinCheck^®^ software, using the grid provided through the software to make calculations (Figure 1). CBCT images were uploaded to Dolphin software and three different points for CBCT data were used per maxillary tooth: Lingual cusp tip of the maxillary posterior teeth, lingual gingival margins (cemento-enamel junction), and lingual cusp tip (Figure 2). These measurements were assessed on both CBCT image and ClinCheck^®^ (Figure 1 and Figure 2). The CBCT measurements were reported to be accurate for this use [18,19]. During posterior maxillary expansion, a combination of posterior teeth crown tipping and bodily expansion can be expected. The analysis of these data was to determine to what degree each (tipping vs. bodily expansion) was represented through this method of expansion. CBCT measurements were similar to Lemos et al. [20]. Paired *t*-tests were performed to show the significance of the changes.

The Shapiro-Wilk test was used to test the assumption of the normality of the continuous data. The paired t-test was used to show the difference between ClinCheck and CBCT measurements. Accurate repeatability of measurements indicated a correlation of 0.95428 between two repeated measures of a random selection of the original measurements.

## 3. Results

A sample of 32 adult subjects were treated with clear aligner maxillary expansion between 1.5 and 3 years. The results of the transverse measurements are portrayed in Table 1. The data show the average predicted movement seen in the ClinCheck^®^ and the actual realized expanded average of CBCT measurements for each tooth at respective points in mm. It should be noted that as the CBCT measurements progress posteriorly, the realized expression of expansion is less than in the more anterior measurements. However, significant expansion was attained (Table 1).

## 4. Discussion

The primary aim of the present study was to evaluate dentoalveolar expansion with Invisalign clear aligners comparing linear measurements in ClinCheck^®^ vs. cone beam computed tomography (CBCT). The differences between these measurements would provide a predictive value of ClinCheck^®^ to final outcomes. Moreover, it would provide an assessment of the reliability of Invisalign ClinCheck^®^ when planning for transverse changes. The extent of the differences between the cups tip widths and CEJ measurements widths attained with the expansion from the Invisalign clear aligners provided information as to buccal tipping and/or bodily translation of the posterior teeth. Limited studies are available comparing ClinCheck^®^ to the final outcome of orthodontic treatment [21]. Three studies [19,22,23] indicated that expansion is possible with Invisalign aligners, but mostly tipping movements [22] and more effective in the premolar area and less effective in the second molar area [23]. One study [21] presented the accuracy of posterior transverse changes with Invisalign, and another indicated that the predictability was reasonable [23]. A sample of adult patients was selected for this study to ensure that transverse growth of the jaws was completed and thus eliminate any doubt that lateral growth could impact the results of this study. Bishara et al. provided the clinician with information showing normal growth data in the arch and including the completion of width dimensions following the completion of the eruption of the permanent dentition [24].

Kravitz et al. [19] measured a 40.5% accuracy of tooth movement during buccal expansion of the anterior teeth. The most accurate movement was lingual constriction. A recommendation was to correct extensive lower incisor irregularity mostly by interproximal reduction (IPR) instead of dentoalveolar expansion. It was also shown that IPR prediction using OrthoCAD closely corresponds with the actual clinical enamel removal by the clinician [25]. This suggestion was provided because retraction utilizing the clear aligners is, for example, more accurate than attained with dentoalveolar expansion of mandibular incisor teeth. The higher accuracy with ClinCheck^®^ in our study may be explained by the fact that the patient sample was provided by a practitioner with extensive experience working with the Invisalign clear aligners system. Moreover, the present study was completed after the publication of the Kravitz et al. study [19]. In addition, the launching of new computer software with modifications in the algorithm and advancements to the material properties (SmartTrack^®^ material) could explain the increase in accuracy of ClinCheck^®^ in our study.

The results of this study showed differences because of the tooth measured and the landmark of measurement. Lateral arch width changes in the maxillary arch were noticed to be 53% correct overall, 62% at the lingual cusp tip, and 42% at the gingival margins. Most accurate predictions were found in the more anterior measurements, and as predictions moved posteriorly, expression of predicted transverse measurements became less. This can be seen with nearly 76% of predicted values of expansion expressed at the first premolar lingual cusp tip and only 44% of predicted values expressed at the mesiolingual cusp of the first molar and 32% at the gingival margin. The greater expansion seemed to occur where the horseshoe aligner provided the most rigidity towards the anterior closed section of the horseshoe. One can speculate that the posterior open section of the U-shaped horseshoe is less rigid and thus, less enabled to provide the expansion needed or predicted.

Overall, the maxillary first molars showed the lowest tracking accuracy. In fact, the measurements showed a trend that ClinCheck^®^ accuracy decreases when moving posteriorly into the arch. This difference is most likely the result of root anatomy, cortical plate thickness, higher mastication loading, and greater soft tissue resistance from the cheeks in the posterior region.

The measurement analysis indicated that greater (over) expansion planned with ClinCheck^®^ was not associated with less accuracy. Moreover, the predicted ClinCheck^®^ tooth movement will not necessarily be more accurate with less expansion. Thus, being aware of these constraints of the appliance reduce errors in tooth movement. According to Phan and Ling (2007), an increase in successful treatment with Invisalign clear aligners when treating narrow dentoalveolar arches by a tipping movement is usually in the range of 0.1 to 5.0 mm [26]. Planned overcorrection during ClinCheck^®^ may have been accomplished with the knowledge that all the expansion could not be obtained as presented.

Two landmarks (cusp tip and gingival margin) were selected in the present study to represent an increase in lateral arch width tipping and bodily movement, respectively. The accuracy of such landmarks was also reported by Baumgaertel et al. [27]. The data of this study suggest that ClinCheck^®^ predicts more bodily movement than Invisalign can actually achieve. As noted in Table 1, expression of expansion was more at cusp tips than at gingival margins, indicating that the tooth achieves its expansion through buccal tipping as well as translation. Our concluding remarks are similar to those of Pavoni et al. (2011), who compared dentoalveolar expansion between Invisalign clear aligners and the Damon system (Ormco, Brea, CA, USA) [2]. Typical expansion, with various types of expanders, is achieved with 18–28% of buccal crown tipping compared to bodily translation of the tooth [20].

It was shown that 70% to 80% of the patients treated with Invisalign clear aligners would require a midcourse modification or a refinement of the treatment plan [28,29]. These numbers indicate a moderately low precision of the expansion treatment planning with ClinCheck^®^. Midcourse corrections or refinements lead to extended treatment time, increased time in the clinic, and larger overhead costs for the orthodontist. Moreover, the latter also lead to higher manufacturing demand for Align Technology. Some of these inaccuracies can be due to clinician inexperience with the technique, the software, or patient non-compliance. Recently, Align introduced the Invisalign G8 SmartForce aligner activation (Align Technology, Align Technology, San Jose, CA, USA), which provides buccal root torque to the maxillary posterior teeth during expansion. This is an important addition to the armamentarium of Invisalign treatment and hopefully will enhance the expansion translation in the future. Treatment precision enhances the result, which makes the need for studies, such as the present research effort on the accuracy of ClinCheck^®^, imperative. The present data may facilitate a reduction of midcourse corrections and refinements. Moreover, the possible prediction of outcome has the potential to assist future practitioners in anticipating the need for overcorrection, thereby reducing refinements, mid-course correction, and treatment time.

The initial arch form prior to the commencement of treatment can be used to determine how much arch expansion can be obtained during treatment. Moreover, it is apparent that teeth in linguoversion or palatoversion might offer more possibilities of expansion and/or overexpansion. This hypothesis would have to be tested in a future study. Overcorrection needs to be included during the planning for expansion on ClinCheck^®^, as well as requesting increased buccal root torque, especially in maxillary premolars and molars. Through-the-bite crossbite elastics may be used to improve the arch width relationship of the teeth. In addition, should expansion not be attainable with clear aligners, the clinician may have to consider conventional expansion modalities prescribed before Invisalign became popular to enhance the success of patient treatment.

## 5. Conclusions

Transverse dental arch expansion can be achieved with Invisalign clear aligners, however, when treatment planning these types of movement, it becomes important to know the limitations of this modality. This study has found that ClinCheck^®^ overpredicts expansion by nearly 30% in the first premolar region and by nearly 70% in the first molar region. It was also shown in this study that more expression of expansion is found at the cusp tips rather than gingival margins, indicating some tipping over bodily translation. Taking these factors into account can help clinicians reduce the need for refinement treatments and midcourse correction, ultimately providing more efficient treatments and better patient experiences. Overcorrecting the ClinCheck^®^ prescription, as well as requesting increased buccal root torque in the posterior, may help to counteract the deficiencies presented with this modality.

## Figures and Tables

**Table 1:** Comparison between transverse measurements of posterior dental arch at T(0) and T(1) using paired T-tests comparing ClinCheck^®^ vs. CBCT measurement.

Variable	ClinCheck^®^ Measurement			CBCT Measurement			Difference			*p*-Value
	N	Mean	Std Dev	N	Mean	Std Dev	N	Mean	Std Dev	
1st Premolar Lingual Cusp	32	4.44	2.35	32	3.38	2.38	32	1.06	1.37	0.0001
1st Premolar Gingiva (CEJ)	32	4.42	2.29	32	2.18	1.85	32	2.24	1.59	<0.0001
2nd Premolar Lingual Cusp	28	4.41	2.43	28	3.04	2.46	27	1.64	1.41	<0.0001
2nd Premolar Gingiva (CEJ)	28	4.35	2.27	28	2.00	1.97	27	2.52	1.43	<0.0001
1st Molar Mesiolingual Cusp	32	4.18	2.04	32	1.83	2.33	32	2.35	1.75	<0.0001
1st Molar Gingiva (CEJ)	32	3.96	1.99	32	1.28	1.41	32	2.68	1.72	<0.0001

## Data Availability

Not applicable.
