# Correction: Santucci et al. Assessment of Posterior Dentoalveolar Expansion with Invisalign in Adult Patients. *Int. J. Environ. Res. Public Health* 2023, *20*, 4318

**DOI:** 10.3390/ijerph20146344

**Published:** 2023-07-11

**Authors:** Vincent Santucci, Paul Emile Rossouw, Dimitrios Michelogiannakis, Tarek El-Bialy, Changyong Feng

**Affiliations:** 1 Private Practice, 1700 Waterfront Building 700, Wichita, KS 67206, USA; 2 Division of Orthodontics and Dentofacial Orthopedics, Eastman Institute for Oral Health, University of Rochester, 625 Elmwood Ave., Rochester, NY 14620, USA; 3 7-020D Katz Group Centre for Pharmacy and Health Research, Department of Dentistry and Dental Hygiene, University of Alberta, Edmonton, AB T6G 2R7, Canada; 4 Medical Center, Department of Biostatistics and Computational Biology, School of Medicine and Dentistry, University of Rochester, Rochester, NY 14642, USA

## Text Correction

There was an error in the original publication [1]. **In the second section, Materials and Methods, the following sentence was incorrect: “The study was reviewed and approved for Ethical and Scientific requirements by the IRB, University of xxx (IRB #xxx).”**.

A correction has been made to *Materials and Methods**, **Paragraph 1**:

**The study was reviewed and approved for Ethical and Scientific requirements by the IRB, University of Rochester (IRB#00003823)**

There was another error in the back matter: **The study was conducted in accordance with the Declaration of Helsinki, and approved by the Institutional Review Board (or Ethics Committee) of the University of Rochester (Study 00003823 on 7 June 2019).”**.

A correction has been made to **Back matter**, **Institutional Review Board Statement**, **Paragraph 1**:

**The study was conducted in accordance with the Declaration of Helsinki and approved by the Institutional Review Board (ethical and scientific requirements) of the University of Rochester (Study 00003823 on 7 June 2019).**

The authors apologize for any inconvenience caused and state that the scientific conclusions are unaffected. The original publication has also been updated.
